# Diagnostic Performance of Artificial Intelligence Models for Periodontitis Disease Detection Using Panoramic Radiographs: A Systematic Review

**DOI:** 10.3390/dj14070416

**Published:** 2026-07-07

**Authors:** Khalid Almutairi, Tariq Almanseer, Enrique España Guerrero, Antonio José España, Gerardo Moreu

**Affiliations:** Department of Stomatology, Section of Periodontology, Faculty of Dentistry, University of Granada, Colegio Máximo s/n, Campus Universitario de Cartuja, 18071 Granada, Spain; khalidalmutiri@correo.ugr.es (K.A.); tareqz@correo.ugr.es (T.A.); antonio@dentalos.es (A.J.E.); gmoreu@ugr.es (G.M.)

**Keywords:** periodontitis, artificial intelligence, machine learning, deep learning, convolutional neural networks, diagnostic accuracy, dental radiography, periodontal diagnosis

## Abstract

**Background/Objectives**: Periodontitis is a highly prevalent inflammatory disease and a major cause of tooth loss worldwide. Accurate diagnosis requires integration of clinical and radiographic findings, but interpretation of panoramic radiographs is subject to variability. Artificial intelligence (AI) has emerged as a promising adjunct for radiographic assessment. This systematic review evaluated the diagnostic performance of AI-based models for detecting periodontitis using panoramic radiographic images. **Methods**: A systematic search of PubMed, Scopus, and Web of Science identified studies published between 1 January 2015 and 1 March 2026. Eligible studies assessed AI models for periodontitis detection on panoramic radiographs and used either clinically confirmed periodontal diagnosis or expert radiographic annotation as the reference standard. Data extraction and quality assessment were performed independently by two reviewers using the QUADAS-2 tool. Owing to heterogeneity in AI architectures, datasets, and outcome measures, a narrative synthesis was conducted. **Results**: Nine studies met the inclusion criteria, comprising more than 20,000 radiographs. AI models included convolutional neural networks (CNNs), segmentation-based systems, and hybrid architectures. Sensitivity ranged from 0.795 to 1.00, specificity from 0.784 to 0.99, and AUC values from 0.843 to 0.967. Studies using clinical periodontal diagnosis as the reference standard generally reported lower performance than those relying solely on expert annotation. Only four studies performed external validation, and dataset sizes varied widely. One study combining panoramic and periapical radiographs showed moderate diagnostic performance. **Conclusions**: AI-based diagnostic models demonstrate promising performance for detecting periodontitis on panoramic radiographs, with several studies reporting high sensitivity and AUC values. However, heterogeneity in reference standards, limited external validation, and inconsistent dataset quality restrict generalizability. AI should be considered an adjunct to, rather than a replacement for, comprehensive clinical periodontal examination. Standardized datasets and robust external validation are needed to support clinical implementation.

## 1. Introduction

Periodontitis is a chronic inflammatory condition that progressively affects the supporting structures of the teeth, including the periodontal ligament, alveolar bone, and gingival tissues, ultimately compromising the stability and integrity of the dentition if left untreated [1]. Oral diseases are highly prevalent worldwide, affecting a large number of individuals and imposing a significant economic burden on society [2].

Periodontitis develops as a result of an imbalance between the resident commensal oral microbiota and the host immune response, leading to oral microbiome dysbiosis, chronic infection, and persistent unresolved inflammation. This process ultimately results in host-mediated destruction of both soft and hard periodontal tissues, including the alveolar bone [3].

The diagnosis of periodontitis is established through both clinical and radiographic examinations. Although diagnosis primarily depends on comprehensive clinical assessment, this process is complex and time-consuming, as it requires evaluation of multiple parameters such as probing pocket depth, clinical attachment loss, and tooth mobility. Consequently, accurate diagnosis demands a high level of clinical expertise [4,5].

This diagnostic complexity highlights the need for adjunctive tools that may assist clinicians in improving consistency and efficiency in periodontal assessment.

Traditionally, periodontitis was classified into chronic (slow-progressing) and aggressive (rapid-progressing) forms based on clinical presentation and extent of tissue destruction. However, the absence of clear pathophysiological distinctions and reliable diagnostic criteria limited this classification. The updated classification system recognizes these forms as part of a spectrum of disease diversity and adopts a multidimensional approach that incorporates various risk factors rather than relying solely on tissue destruction [6].

In recent years, artificial intelligence (AI) techniques have demonstrated considerable potential in the development of diagnostic systems for periodontitis. AI in dentistry encompasses multiple methodological approaches, including machine learning, deep learning, convolutional neural networks (CNNs), and image-based segmentation models, each differing in complexity and learning capability [7]. CNNs, in particular, are the most widely applied in dental radiographic analysis due to their strong capability in image interpretation and automatic feature extraction. These models have been increasingly investigated for detecting periodontal disease using panoramic radiographs [8]. This body of evidence is primarily based on panoramic radiographic imaging. Despite the growing number of studies investigating artificial intelligence for periodontal diagnosis, the reported diagnostic performance varies across different models, datasets, and validation strategies, leading to heterogeneity in the available evidence. Therefore, a comprehensive synthesis of the current literature is required to better understand the overall diagnostic accuracy and clinical applicability of these models.

Beyond diagnostic imaging, recent advances in generative AI have expanded the role of artificial intelligence in dentistry beyond image-based applications. These systems have shown potential in clinical decision support, patient communication, education, and research workflows [9].

Therefore, the aim of this systematic review is to evaluate the diagnostic performance of AI-based models for detecting periodontitis using panoramic radiographs and to compare their accuracy and effectiveness with conventional clinical and radiographic diagnostic methods performed by dental professionals.

## 2. Materials and Methods

### 2.1. Study Protocol and Focused Question

This systematic review was prepared in accordance with the Preferred Reporting Items for Systematic Reviews and Meta-Analyses (PRISMA) guidelines [10]. The review protocol was prospectively registered in the International Prospective Register of Systematic Reviews (PROSPERO), maintained by the Centre for Reviews and Dissemination, University of York, United Kingdom, under the registration number CRD420261361075. The PRISMA 2020 checklist is provided as Supplementary Materials (Table S1). The protocol predefined the focused question, eligibility criteria, outcomes of interest, and data synthesis methods prior to study screening, which included studies published between 1 January 2015 and 1 March 2026, and no deviations from the registered protocol occurred.

The review aimed to address the following focused question based on the PICOS framework:

In human subjects diagnosed with periodontitis, what is the diagnostic performance of artificial intelligence-based models, including machine learning, deep learning, convolutional neural networks, and segmentation techniques, compared with conventional clinical diagnostic and expert annotation methods performed by dental professionals, in terms of diagnostic accuracy, sensitivity, specificity, precision, recall, and area under the curve (AUC), in original research studies published between 2015 and 2026?

### 2.2. Eligibility Criteria

Eligibility criteria were defined according to the PICOS framework.

**Inclusion criteria:** Original research studies evaluating artificial intelligence (AI) models for the detection of periodontitis using panoramic radiographic images in human subjects. Eligible studies included those that used either clinically confirmed periodontitis diagnosed through conventional periodontal examination or expert radiographic annotation performed by experienced dental professionals as the reference standard. Studies reporting diagnostic performance outcomes such as accuracy, sensitivity, specificity, precision, recall, or area under the curve (AUC) were included. Only studies published in peer-reviewed journals in English between 2015 and 2026 were considered.

**Exclusion criteria:** Reviews, systematic reviews, meta-analyses, case reports, editorials, letters, and conference abstracts were excluded. Animal and in vitro studies were excluded. Studies not using AI for diagnostic purposes. Studies not using panoramic radiographic images were excluded.

### 2.3. Information Sources and Search Strategy

A comprehensive electronic search was conducted in PubMed, Scopus, and Web of Science to identify relevant studies. The search included both MeSH terms and free-text keywords related to periodontitis, artificial intelligence, machine learning, deep learning, convolutional neural networks, segmentation, and diagnostic accuracy. Boolean operators (AND, OR) were used to combine search terms. The search was limited to human studies published in English between 1 January 2015 and 1 March 2026. Studies not using panoramic radiographs or not reporting diagnostic performance metrics were excluded.

### 2.4. Search Strategy

A comprehensive literature search was conducted by KA and TA to identify relevant studies related to the topic of this review. The following electronic databases were searched: Scopus, PubMed, and Web of Science. The search strategy was applied using predefined keywords and Boolean operators. The search was limited to articles published in English between 1 January 2015 and 1 March 2026. No restrictions were applied regarding study design in order to ensure a broad and comprehensive inclusion of relevant literature. In addition, a manual search of the reference lists of eligible studies was performed to identify further relevant articles. Detailed search strategies for each database are provided in the Supplementary Materials (Table S2).

### 2.5. Data Extraction

Data extraction was performed independently by two reviewers using a standardized data extraction form. Extracted data included study characteristics, population details, AI model type, imaging modality, and diagnostic performance metrics. In cases of missing or unclear data, study authors were contacted for clarification. Disagreements were resolved through consensus. For studies with multiple reports, only relevant, non-duplicated data were included.

### 2.6. Quality Assessment (Risk of Bias)

The methodological quality and risk of bias of included studies were assessed using the QUADAS-2 tool for diagnostic accuracy studies. This tool evaluates four domains: patient selection, index test, reference standard, and flow and timing. Each domain was rated as low, high, or unclear risk of bias. Assessments were performed independently by two reviewers, and disagreements were resolved through discussion or consultation with a third reviewer. Results were summarized in tabular form and used to interpret study reliability.

### 2.7. Data Synthesis and Statistical Analysis

Data from included studies were systematically organized to evaluate the diagnostic performance of artificial intelligence (AI) models in detecting periodontitis using panoramic radiographs. A narrative synthesis was conducted due to heterogeneity in study designs, AI models, imaging protocols, and outcome measures across the included studies. Results were summarized and compared descriptively according to AI model type, study characteristics, and reported diagnostic performance metrics. The overall certainty of the evidence was considered when interpreting the findings.

## 3. Results

### 3.1. Study Selection

**Figure 1:**
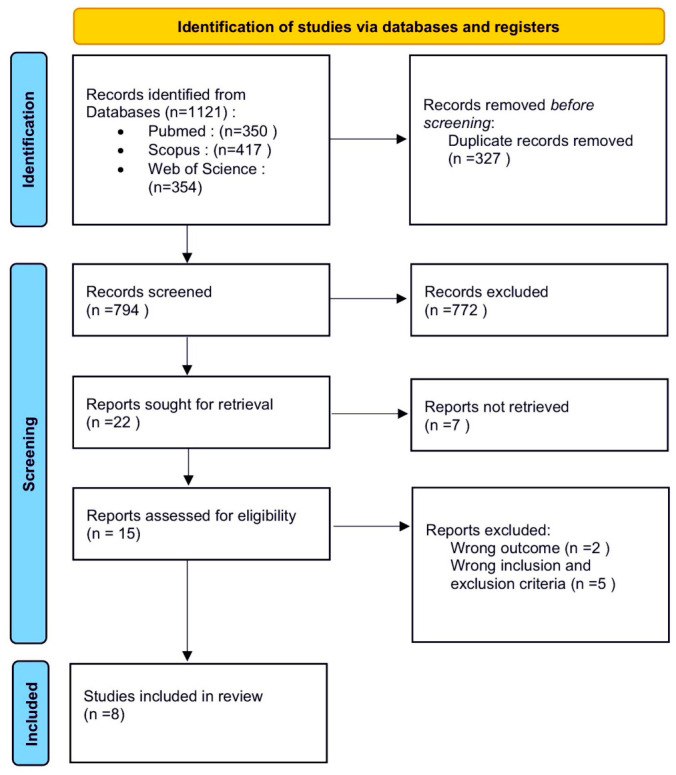
Flowchart summarizing the results of the selection process (PRISMA 2020).

A flowchart illustrating the study selection process was created in accordance with the PRISMA 2020 guidelines to provide a clear visual summary of the screening steps. This study selection process is illustrated in the PRISMA 2020 flow diagram presented in Figure 1. Using this process, a total of 1122 records were initially retrieved through the electronic database search. After removing duplicates and screening the titles and abstracts, 772 studies were assessed for relevance. Following full-text evaluation against the predefined eligibility criteria, 15 reports were assessed for eligibility. After full-text evaluation, 7 studies were excluded for reasons such as wrong outcome and inappropriate eligibility criteria. Finally, 8 studies were included in this systematic review. This systematic approach ensured that only studies meeting the inclusion criteria were selected, thereby enhancing the rigor and reliability of the review.

### 3.2. Study Characteristics and Interventions

The included studies demonstrated substantial heterogeneity in study design, dataset composition, artificial intelligence (AI) architecture, and validation strategies, all of which influenced the reported diagnostic performance. Most studies adopted retrospective designs utilizing panoramic radiographs, with dataset sizes ranging from 6 to over 12,000 radiographic images, with some studies including more than 45,000 annotated regions of interest. A variety of advanced deep learning architectures were employed, including single-stage detectors such as YOLOv8, two-stage detectors such as Faster R-CNN and Cascade R-CNN, and hybrid approaches integrating convolutional neural networks with traditional machine learning classifiers such as support vector machines [11,12,13,14,15,16,17,18].

Performance differences were observed according to the methodological approach, as studies employing segmentation-based techniques demonstrated superior performance due to their ability to localize anatomical landmarks and quantify radiographic bone loss. Similarly, studies utilizing detection–classification pipelines also showed enhanced diagnostic capability [13,17,18].

Regarding reference standards, a clear distinction emerged between studies relying on expert annotation and those incorporating clinical diagnosis. Studies that used periodontists as the gold standard and, in some cases, integrated clinical examination data provided stronger clinical validity compared with annotation-only studies [13,14,15].

Additionally, studies incorporating multi-expert consensus improved diagnostic reliability by reducing inter-observer variability. In contrast, studies based solely on image annotation, despite demonstrating high internal performance metrics, may overestimate real-world applicability [14,15,16].

Comparative analyses between AI systems and clinicians were limited but informative, as some studies reported comparable or superior diagnostic agreement of AI relative to clinicians. Furthermore, studies incorporating external validation demonstrated better assessment of model generalizability compared with those relying exclusively on internal validation [11,12,15,18].

### 3.3. Effects of Intervention: Outcomes

The characteristics of the included studies and the diagnostic performance of the AI models are summarized in Table 1.

#### 3.3.1. Accuracy

Across the included studies, artificial intelligence (AI) systems demonstrated high diagnostic accuracy, although variability was observed depending on study design and reference standards. Models evaluated against expert annotation generally achieved the highest accuracy, reflecting strong agreement between AI predictions and labeled anatomical structures. In contrast, studies incorporating clinically confirmed diagnoses reported slightly lower but more clinically representative performance [15]. Additionally, studies including external validation showed a modest decline in accuracy compared with internal validation, highlighting the impact of dataset heterogeneity while supporting model generalizability [11,15].

#### 3.3.2. Sensitivity

Sensitivity values across the included studies ranged from 0.70 to 1.00, indicating a high ability of AI models to correctly identify periodontal disease. However, one study reported substantially reduced specificity during clinical implementation, suggesting a tendency toward false-positive predictions despite excellent detection capability [13]. Near-ceiling sensitivity values reaching up to 0.999 were demonstrated in some studies, while another study reported sensitivity values of up to 0.956. Additionally, one study demonstrated a sensitivity of 0.795 [11,16,17].

#### 3.3.3. Specificity

Specificity values ranged from 0.73 to 1.00 across the included studies, reflecting variability in the ability of AI models to correctly identify non-diseased cases. Although one study demonstrated specificity of 0.98 in the segmentation phase, the clinical implementation phase reported specificity of 0%, indicating a substantial trade-off between increase in false-positive detection [13]. Another study demonstrated specificity values of up to 0.954, while one study reported a specificity of 0.784, closely aligning with expert-level diagnosis [11,15].

#### 3.3.4. Precision

Precision-related performance, as reflected in F1-scores and model behavior, varied across the included studies. One study reported an F1-score of 0.90 during segmentation model evaluation, indicating strong balance between precision and recall at the anatomical detection level [13]. Studies based on expert annotation demonstrated very high precision, with F1-scores reaching up to 0.996. Similarly, one study reported F1-scores of up to 0.97 in segmentation tasks. These findings suggest that models trained on high-quality annotated datasets tend to achieve higher precision [14,16,17].

#### 3.3.5. Recall

Recall values were consistently high across the included studies, with several models achieving optimal performance. One study reported perfect recall (1.00) in binary classification tasks, indicating excellent detection capability. However, in more complex tasks such as severity classification within the same study, recall decreased to 0.74, reflecting increased task difficulty [18]. Overall, recall values across studies reached up to 1.00, particularly in models optimized for detection tasks [18].

#### 3.3.6. AUC

The area under the curve (AUC) values across the included studies ranged from 0.843 to 0.967, indicating strong overall diagnostic performance. One study reported AUC values between 0.942 and 0.967, while another study demonstrated AUC values of 0.843 for internal validation and 0.793 for external validation [11,15]. Another study achieved an AUC value of 0.91, indicating strong diagnostic performance in panoramic radiograph analysis [14]. Overall, AI models demonstrated consistently high diagnostic performance for periodontal disease detection on panoramic radiographs across different study designs and validation approaches [11,14,15].

### 3.4. Risk of Bias Assessment

Across the nine included studies, the overall risk of bias demonstrated notable variability across the four QUADAS-2 domains, with several recurring methodological limitations. Patient selection was the most frequently affected domain, with the majority of studies exhibiting either high risk of bias or some concerns. This was primarily attributed to retrospective study designs, single-center data sources, and the application of strict inclusion and exclusion criteria. Studies such as [12,13,15,16] were judged to have a high risk of bias in this domain, mainly due to non-random sampling and exclusion of specific cases (e.g., poor-quality images or discordant diagnoses), which may have resulted in non-representative datasets. Similarly, refs. [14,17] demonstrated moderate concerns, while ref. [11] raised concerns related to convenience sampling and heterogeneity in disease severity. These limitations collectively reduce the external validity and generalizability of the findings [11,12,13,14,15,16,17]. Regarding the index test domain, most studies were judged as having unclear risk of bias or some concerns. This was largely due to insufficient reporting of blinding procedures and lack of clarity on whether model thresholds were pre-specified prior to evaluation. Studies such as [13,15,16] did not clearly report blinding, increasing the risk of performance overestimation. One study showed a high risk of bias due to potential overfitting and lack of transparency in model tuning. In contrast, another study demonstrated a low risk of bias in this domain, as the AI model was clearly defined and reproducible. Overall, inadequate methodological transparency was a common issue affecting this domain [13,15,16,17,18]. The reference standard domain showed comparatively better methodological quality across studies, although some important limitations were still identified. Low risk of bias was reported in studies such as [12,14,15,18], where diagnoses were based on expert evaluation, often supported by clinical and radiographic findings. However, other studies, including [13], demonstrated a high risk of bias due to exclusion of discordant cases or reliance on less robust diagnostic criteria, potentially inflating diagnostic performance. Another study also showed moderate risk due to reliance on radiographs alone without clinical confirmation, while one study presented concerns due to the use of mixed and heterogeneous diagnostic standards [12,13,14,15,17,18]. In the flow and timing domain, the risk of bias was generally lower compared to other domains; however, several studies still presented important concerns. Low risk of bias was observed in [12,16], where consistent evaluation procedures were applied. In contrast, two studies demonstrated high risk of bias due to inconsistent application of the reference standard and exclusion of cases, which may have introduced bias. Other studies showed moderate or unclear risk due to lack of external validation, variability in datasets, or insufficient reporting of timing between index and reference tests [12,13,14,15,16,17,18]. In summary, the overall methodological quality of the included studies ranged from moderate to high risk of bias, with patient selection and index test domains representing the most critical sources of bias. Common issues included retrospective designs, lack of blinding, exclusion of discordant cases, and insufficient reporting of AI model development and validation processes. While the reference standard domain was relatively robust in several studies, inconsistencies in diagnostic criteria and reliance on non-clinical standards in some cases remain a concern. These limitations should be carefully considered when interpreting the findings of this systematic review, particularly in terms of the generalizability and clinical applicability of AI-based diagnostic models in dentistry [11,12,13,14,15,16,17,18].

**Figure 2:**
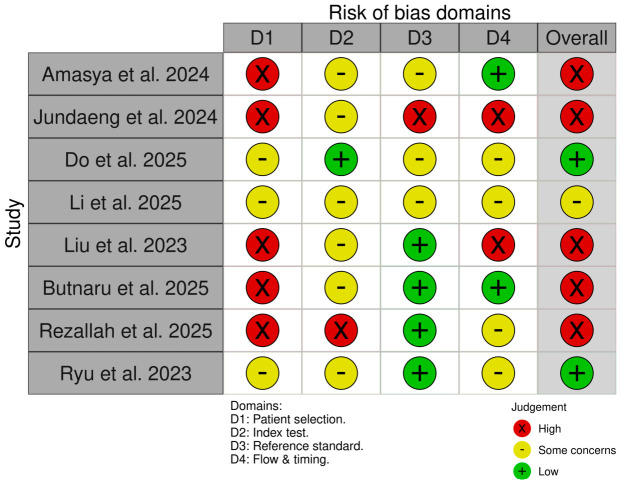
Summary of risk of bias assessment for the included studies using the QUADAS-2 tool. Each domain (patient selection, index test, reference standard, and flow and timing) is presented for all included studies, with judgments categorized as low, high, or unclear risk of bias [11,12,13,14,15,16,17,18].

**Figure 3:**
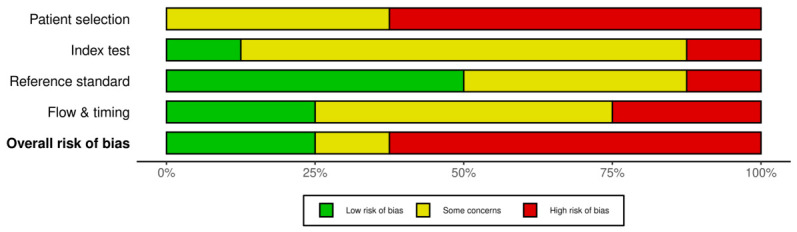
Distribution of risk of bias across the included studies according to the QUADAS-2 tool. The bar chart illustrates the proportion of studies with low, high, and unclear risk of bias within each domain, providing an overall overview of methodological quality.

Therefore, although AI-based diagnostic models in dentistry demonstrate promising performance, the presence of methodological limitations and potential biases across the included studies warrants cautious interpretation of the findings, highlighting the need for well-designed prospective, multicenter studies with standardized protocols to ensure more reliable and generalizable evidence [11,12,13,14,15,16,17,18]. Overall, the findings presented in Figure 2 and Figure 3 indicate that patient selection and index test were the domains most frequently associated with methodological concerns, whereas the reference standard and flow and timing domains generally showed lower risks of bias.

## 4. Discussion

### 4.1. Overall Diagnostic Performance of AI Models

The present systematic review demonstrates that artificial intelligence (AI) models achieve consistently high diagnostic performance in the detection and assessment of periodontal disease across a wide range of study designs, datasets, and methodological approaches. Despite the observed heterogeneity in AI architectures, dataset composition, and validation frameworks, the overall findings indicate that AI systems are capable of delivering clinically competitive results, particularly when trained and validated using high-quality reference standards.

### 4.2. Influence of Reference Standards on Diagnostic Performance

A key finding of this review is the influence of the reference standard on reported performance. Studies relying primarily on expert annotation consistently reported the highest diagnostic metrics, including near-ceiling sensitivity and precision [14,16,17]. This strong performance likely reflects the high agreement between AI predictions and well-defined anatomical labels. However, such results may overestimate real-world clinical applicability, as annotation-based ground truth does not fully capture the complexity and variability of clinical diagnosis. In contrast, studies incorporating clinically confirmed diagnoses demonstrated slightly lower but more realistic performance, highlighting the importance of clinically relevant validation frameworks [15]. This distinction emphasizes the need for careful interpretation of AI performance based on the reference standard used.

### 4.3. Impact of Methodological Design and Model Architecture

Another important observation is the impact of methodological design on AI performance. Segmentation-based models and detection–classification pipelines consistently outperformed classification-only approaches [13,17,18]. This improvement is attributed to their ability to localize anatomical structures and quantify radiographic bone loss, thereby enhancing both diagnostic accuracy and interpretability. In several detection tasks, very high sensitivity values were reported; however, these were occasionally associated with reduced specificity, indicating a potential trade-off between disease detection and false-positive rates [13]. This suggests that maximizing sensitivity alone may not always ensure optimal clinical utility.

### 4.4. Comparison Between AI Systems and Clinicians

The comparison between AI systems and clinicians, although limited to a small number of studies, provides important clinical insight. AI performance was generally comparable to periodontal specialists and, in some cases, superior to general practitioners and less experienced clinicians [13,15]. Similarly, a deep learning model for periodontal bone loss detection using panoramic radiographs achieved a higher F1-score than experienced dental clinicians, supporting the potential role of AI as a reliable clinical decision-support tool in periodontal diagnosis [19]. However, most comparisons were conducted under experimental conditions rather than routine clinical practice, highlighting the need for further prospective clinical studies before widespread implementation. Additionally, recent evidence suggests that AI-based systems applied to panoramic radiographs and intraoral images may serve as effective screening and triaging tools, particularly in primary care and tele dentistry settings where access to advanced imaging modalities such as CBCT is limited [20,21].

### 4.5. Influence of Imaging Modality

Another important consideration is the imaging modalities used across the included studies. CBCT provides volumetric information that allows AI models to evaluate complex periodontal structures in three dimensions [22]. In contrast, panoramic radiographs offer wide coverage but present inherent limitations such as anatomical overlap, image distortion, magnification errors, and reduced resolution compared with periapical radiographs [23,24,25,26]. These factors may affect both clinician interpretation and AI-based diagnosis.

### 4.6. Advances in Deep Learning Approaches

CNN models trained on panoramic images have achieved accuracies ranging from 0.80 to 0.84, with relatively balanced sensitivity and specificity [24,25,26]. Despite these limitations, high diagnostic performance can still be achieved using advanced deep learning strategies. Recent studies using standardized imaging workflows and modern CNN, transformer, or hybrid architectures have demonstrated improved performance in controlled settings [27,28]. These improvements are mainly attributed to feature fusion and multi-architecture integration, even when working with relatively small datasets [23]. These findings are consistent with the present review, where segmentation-based models and multi-stage deep learning pipelines consistently outperformed simpler classification approaches. Furthermore, combining multiple architectural components has been shown to significantly enhance model robustness and generalization in complex imaging modalities such as panoramic radiographs [23]. Recent work has also highlighted the promise of AI for photographic detection of periodontal disease, while emphasizing the need for standardized acquisition protocols and multicenter datasets to ensure reproducibility and clinical translation [20].

### 4.7. External Validation and Generalizability

External validation emerged as a critical factor influencing model performance and generalizability. Studies incorporating external datasets consistently demonstrated a modest decline in performance compared to internal validation [11,15]. This finding reflects differences in imaging conditions, patient populations, and annotation protocols across datasets. Nevertheless, the relatively stable performance observed under external validation supports the potential of these models for real-world clinical application. These findings further emphasize the importance of multicenter datasets and standardized validation protocols to improve the robustness and transferability of AI systems across different clinical environments.

### 4.8. Limitations of the Available Evidence

Despite the promising findings, several limitations were identified across the included studies. Variability in ground truth definition, differences in evaluation levels (tooth-, image-, or patient-level), and the lack of standardized validation frameworks limit direct comparability between studies. In addition, the predominance of retrospective study designs and reliance on internal validation may introduce bias and reduce the robustness of reported performance metrics. The relatively small number of eligible studies and methodological heterogeneity further limit the strength of the evidence.

### 4.9. Future Directions and Clinical Implications

Therefore, while AI-based diagnostic systems demonstrate strong potential in periodontology, the current evidence should be interpreted with caution until validated by larger prospective studies using standardized methodologies and clinically robust reference standards.

## 5. Conclusions

Within the limitations of the included studies, AI systems demonstrate high diagnostic performance in the detection and assessment of periodontal disease, with sensitivity, specificity, and AUC values indicating clinically competitive results. Models trained on expert-annotated datasets achieve the highest performance, while those validated against clinical diagnosis provide more realistic estimates of real-world applicability.

Segmentation-based and multi-stage AI approaches appear to offer superior performance compared to classification-only models, particularly in tasks requiring anatomical localization and disease severity assessment. Although external validation results indicate a modest reduction in performance, they confirm the generalizability of AI systems across heterogeneous datasets.

Future research should focus on the standardization of validation protocols, the integration of clinical diagnostic criteria, and the expansion of externally validated datasets to enhance the reliability and clinical translation of AI-based diagnostic systems in periodontology.

## Figures and Tables

**Table 1:** (**A**) Characteristics of included studies. (**B**) Diagnostic performance of AI models.

(**A**)							
**Author (Year)**	**Country**	**Sample Size (Radiographs)**	**Imaging Modality**	**Study Design**	**Reference Standard**	**Dataset Split**	**External Validation**
Li et al. (2025) [11]	China	12,023	OPG	Retrospective AI development study	Clinical periodontal examination + specialist consensus	Internal + external test cohorts	Yes
Butnaru et al. (2025) [12]	Romania	6 (pilot radiographs)	OPG	Pilot diagnostic AI study	Expert reader annotation	80/20 train-validation (reported)	Yes
Jundaeng et al. (2025) [13]	Thailand	2000	OPG	Retrospective deep learning study	Periodontist + general practitioner consensus	70/10/20 split	No
Ryu et al. (2023) [14]	South Korea	100	OPG	Retrospective CNN study	Expert annotation	5-fold cross-validation	No
Liu et al. (2024) [15]	China	2275	OPG	Diagnostic classification study	Expert + GP annotation	1267/376/272 split	Yes
Amasya et al. (2023) [16]	Turkey	3200 teeth/4896 surfaces	OPG	Segmentation and detection study	Expert annotation	Not reported	No
Do et al. (2023) [17]	England	500	OPG	Automated DL diagnostic study	Expert annotation	75/15/10 split	No
Rezallah et al. (2024) [18]	UAE	817	OPG	Comparative AI model study	Expert validation	80/20 + external test (n = 600)	Yes
(**B**)							
**Author (Year)**	**AI Model**	**Sensitivity**	**Specificity**	**Accuracy**	**AUC**	**F1-Score**	**Recall**
Li et al. (2025) [11]	HC-Net/hybrid CNN	0.956	0.826	NR	0.967	NR	0.902–0.956
Butnaru et al. (2025) [12]	Embedded CNN (Planmeca Romexis AI)	NR	NR	NR	NR	NR	NR
Jundaeng et al. (2025) [13]	Deep learning classification model	1.00	0.00	NR	NR	0.90	1.00
Ryu et al. (2023) [14]	CNN	0.91	NR	NR	0.91	0.90	0.91
Liu et al. (2024) [15]	AlexNet-based CNN	0.795	0.784	NR	0.843 (internal)/0.793 (external)	NR	0.795
Amasya et al. (2023) [16]	CNN segmentation model	0.904–0.999	NR	NR	NR	0.985	0.904–0.999
Do et al. (2023) [17]	Deep learning model	0.94	0.99	NR	NR	0.98	0.94
Rezallah et al. (2024) [18]	MobileNetV2/YOLOv8	1.00/0.70	NR	NR	NR	NR	1.00/0.74

## Data Availability

No new data were created or analyzed in this study. Data sharing is not applicable to this article.

## Supplementary Materials

The following supporting information can be downloaded at: https://www.mdpi.com/article/10.3390/dj14070416/s1, Table S1: PRISMA 2020 Checklist; Table S2: Search strategy used in databases.

## Abbreviations

The following abbreviations are used in this manuscript.

AI	Artificial Intelligence
AUC	Area Under the Receiver Operating Characteristic Curve
CNN	Convolutional Neural Network
CAL	Clinical Attachment Level
CEJ	Cemento–Enamel Junction
BOP	Bleeding on probing
OPG	Orthopantomogram (panoramic radiograph)
RBL	Radiographic Bone Loss
PDL	Periodontal Ligament
STARD	Standards for Reporting Diagnostic Accuracy Studies
IRB	Institutional Review Board
BCE	Binary Cross Entropy
MSE	Mean Squared Error
CAM	Class Activation Map
SPSS	Statistical Package for the Social Sciences
ICC	Intraclass Correlation Coefficient
CI	Confidence Interval
SD	Standard Deviation
NR	Not Reported
FP	False Positive
TN	True Negative
FN	False negative
Sens	Sensitivity
Spec	Specificity
PPV	Positive Predictive Value
NPV	Negative Predictive Value
F1	F1 Score
ROC	Receiver Operating Characteristic
DL	Deep Learning
HC-Net	Hybrid Classification Network
SUR	Sapienza University of Rome
ANOVA	Analysis of Variance
PER	Periapical Radiograph
